# Quantitative Estimate Index for Early-Stage Screening of Compounds Targeting Protein-Protein Interactions

**DOI:** 10.3390/ijms222010925

**Published:** 2021-10-10

**Authors:** Takatsugu Kosugi, Masahito Ohue

**Affiliations:** Department of Computer Science, School of Computing, Tokyo Institute of Technology, G3-56-4259 Nagatsutacho, Midori-ku, Yokohama 226-8501, Kanagawa, Japan; kosugi@li.c.titech.ac.jp

**Keywords:** drug discovery, protein-protein interaction (PPI), PPI-targeting drug, virtual screening, QED, QEPPI

## Abstract

Drug-likeness quantification is useful for screening drug candidates. Quantitative estimates of drug-likeness (QED) are commonly used to assess quantitative drug efficacy but are not suitable for screening compounds targeting protein-protein interactions (PPIs), which have recently gained attention. Therefore, we developed a quantitative estimate index for compounds targeting PPIs (QEPPI), specifically for early-stage screening of PPI-targeting compounds. QEPPI is an extension of the QED method for PPI-targeting drugs that models physicochemical properties based on the information available for drugs/compounds, specifically those reported to act on PPIs. FDA-approved drugs and compounds in iPPI-DB, which comprise PPI inhibitors and stabilizers, were evaluated using QEPPI. The results showed that QEPPI is more suitable than QED for early screening of PPI-targeting compounds. QEPPI was also considered an extended concept of the “Rule-of-Four” (RO4), a PPI inhibitor index. We evaluated the discriminatory performance of QEPPI and RO4 for datasets of PPI-target compounds and FDA-approved drugs using F-score and other indices. The F-scores of RO4 and QEPPI were 0.451 and 0.501, respectively. QEPPI showed better performance and enabled quantification of drug-likeness for early-stage PPI drug discovery. Hence, it can be used as an initial filter to efficiently screen PPI-targeting compounds.

## 1. Introduction

Protein-protein interactions (PPIs) have attracted attention as drug targets since the early 2000s [1,2,3,4,5]. However, it is difficult to design drugs for PPIs based on conventional rules, such as Lipinski’s rule of five (RO5) [6,7], because their physicochemical characteristics are very different from those of conventional drug targets [8,9]. In fact, only a few PPI inhibitors have been approved to date, and few PPI-targeting drug candidates have advanced in clinical trials to subsequent phases [10]. Therefore, an index that can be used to computationally select compounds that are likely to target PPIs is needed.

The quantitative estimate of drug-likeness (QED), which was proposed in 2012 [11], is an index of drug-likeness modeled using information available on marketed drugs and is widely used in current small-molecule drug discovery for computational methods [12,13] and to evaluate drug-like properties [14]. The QED index models these properties using data available from 771 orally administered drugs already approved by the U.S. Food and Drug Administration (FDA). However, this index is not an appropriate measure for PPI-targeting compounds, which require a relatively large surface area of the protein with which to interact. Therefore, new measures should be developed for PPI-targeting drugs [15].

QEX [16] and QEPT [17] are examples of QED remodeling methods that are based on the concept of QED and involve modeling physicochemical properties. In the case of QEX, the target compounds act on each target protein. In the case of QEPT, the target compounds are organic chemicals obtained from plant roots. These compounds represent quintessential successful models for evaluating physicochemical properties. The idea is based on the remodeling of a PPI-targeting drug based on an already approved PPI-targeting drug. However, many molecular optimizations must be performed before approval. Even PPI-targeting compounds have been optimized to exhibit the general characteristics of drugs, such as RO5 (low-molecular-weight and water solubility). However, indices such as QED are mainly used in the early stages of drug discovery, that is, seed compound discovery. The metrics modeled from PPI-targeting drugs that are already available on the market are idealistic and unsuitable for the early stage.

In recent years, small-molecule de novo design methods have increasingly been used in combination with generative modeling and reinforcement learning [12,13,18,19]. However, it is difficult to design PPI-targeting compounds using these methods, and there is currently no continuous index of the likelihood of PPI-targeting compounds.

In this study, we developed a method named QEPPI (Quantitative Estimate Index for Compounds Targeting Protein-Protein Interactions), which is useful for early-stage PPI-targeting drug discovery based on data from compounds that have undergone extensive PPI inhibition or stabilization experiments, rather than on data from marketed PPI-targeting drugs. The code and easy-to-use environment of QEPPI are available on Google Colaboratory at https://github.com/ohuelab/QEPPI, accessed on 30 September 2021.

We published a preliminary version of this work in conference proceedings [20], including only limited experiments and discussion. In the current study, we have expanded the introduction and a discussion based on the results of physicochemical characterization of PPI modulators by Truong et al. [21]. In addition, we developed an implementation code to make QEPPI publicly available.

## 2. Results

### 2.1. Model Building for QEPPI

QEPPI is an index in the early-stages of PPI drug discovery, and the prerequisites for using the dataset to model QEPPI are as follows:

- Not limited to those in the approval phase or marketed after approval, as various optimizations will be performed during the approval phase.
- Not limited to PPI structures or complexes of protein and PPI-targeting compounds with known structures.

The reason for these requirements is that a drug undergoes many molecular optimizations before it is approved. Therefore, if only approved compounds are used as the dataset to create the model, too many ideal compounds will be identified as unsuitable for this purpose. In addition, data on various candidate compounds are necessary for the initial stage of the search. According to a recent review by Shin et al. [15], if only compounds with known structures are selected for X-ray crystallography of proteins and ligands, the amount of data that can be handled will involve tens to hundreds of compounds, although more than 720,000 human PPIs are known BioGrid [22] Current Build Statistics (4.3.196)—April 2021). Considering the number of known PPIs, structural information on PPIs likely remains insufficient. We hypothesized that information on the target proteins and their ligand compounds would be sufficient without requiring three-dimensional structures.

Therefore, we used iPPI-DB [23], which was manually curated from the literature. In total, 2361 PPI-targeting compounds are registered in this database (as of 21 April 2021), which are primarily derived from PPI inhibition or stabilization experiments. The number of compounds registered in DrugBank was 43, which is approximately 1.8% of the total. The quality and quantity of the data meet the requirements of the dataset for modeling QEPPI.

**Figure 1:**
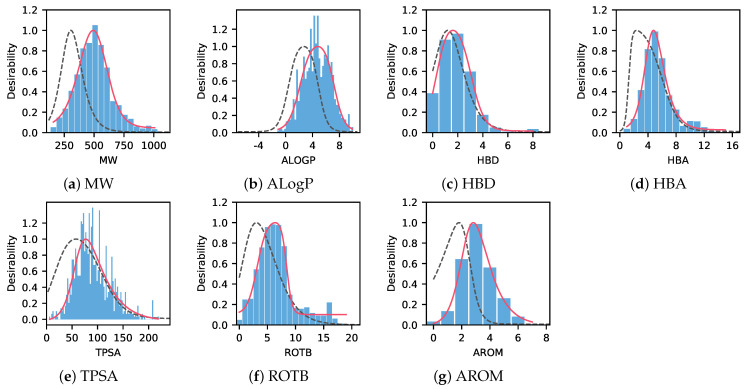
Histograms of seven molecular physicochemical properties for a set of non-redundant compounds of iPPI-DB. Molecular weight (MW) (**a**), LogP value estimated by Ghose-Crippen method (ALogP) (**b**), number of hydrogen bond donors (HBD) (**c**), number of hydrogen bond acceptors (HBA) (**d**), topological molecular polar surface area (TPSA) (**e**), number of rotatable bonds (ROTB) (**f**), and number of aromatic rings (AROM) (**g**). The solid red lines describe the asymmetric double sigmoid (ADS) function (1) used to model the QEPPI histograms. The black dashed lines describe the ADS function used to model the quantitative estimate of drug-likeness (QED) histograms.

We built the QEPPI model using data selected after clustering for non-redundancy (see Methods for details). The histograms of the distributions of seven molecular physicochemical properties, MW, ALogP, HBD, HBA, TPSA, ROTB, and AROM, are shown in Figure 1. The distribution peaks and optimized weights $w_{i}$ of each physicochemical property are shown in Table 1.

Figure 1 and Table 1 show that oral drugs and PPI-targeting compounds have very different properties. Table 1 shows that the peak values of all properties were higher for QEPPI than for QED. Particularly, the major difference between QEPPI and QED is the peak value of ALogP (QEPPI: 4.78, QED: 2.70), suggesting that low lipophilicity and high hydrophilicity are important for oral drugs in terms of oral absorption. This suggests that QEPPI can capture PPI-targeting drug-like properties compared to QED and has a different role in the seed compound discovery process, which is the early-stage of drug discovery.

### 2.2. Evaluation of QEPPI

**Figure 2:**
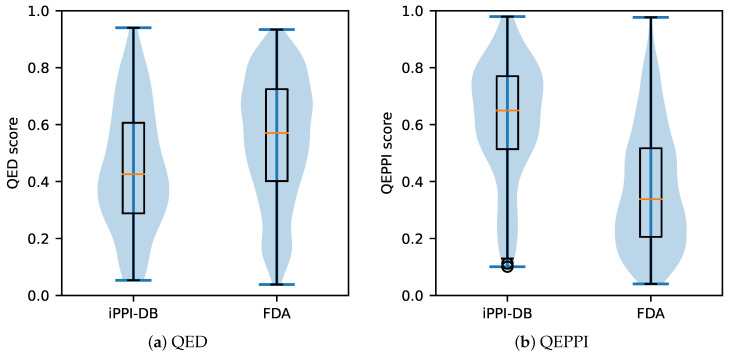
Distribution of QED and QEPPI in the PPI-targeting compounds dataset and FDA-approved drug dataset. Each filled area extends to represent the entire data range, with optional lines at the median. The QED score was calculated for both datasets (**a**). The QEPPI score was calculated for both datasets (**b**).

To evaluate whether QEPPI, which was developed in this study, is a more useful index for early-stage PPI drug discovery compared to QED, we obtained data on 321 PPI-targeting compounds from the iPPI-DB that were not used for model building (iPPI-DB dataset). In addition, we obtained data on 1596 FDA-approved drugs, excluding duplicates and approved drugs targeting PPI (FDA dataset). The QED score was calculated using these data; the distribution of these values is shown in Figure 2a. Similarly, the QEPPI score was calculated, and the distribution of the values is shown in Figure 2b.

**Figure 3:**
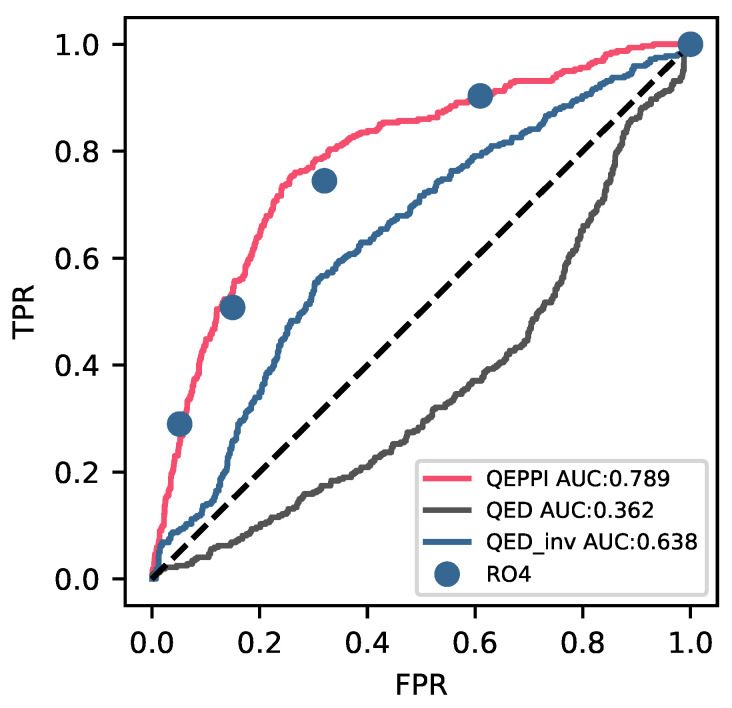
Comparison of QEPPI with other measures of drug-like properties in receiver operating characteristic (ROC) curves. All ROC curves show that the true-positive rate against the false-positive rate describes the differences in performance for classifying compounds as PPI-targeting compounds. The red, black, and blue lines represent the ROC curves for QEPPI, QED, and 1−QED (QED_inv), respectively. The five blue dots are plotted as points that allowed 0 to 4 violations of RO4. The dashed black line represents a random prediction of the dataset.

Figure 2a shows that PPI-targeting compounds exhibit a lower distribution of QED scores compared to conventional drugs, suggesting that QED is not an appropriate measure for PPI-targeting compounds, as it typically represents oral drug-like properties rather than drug-likeness. Figure 2b shows that PPI-targeting compounds have a higher distribution of QEPPI scores compared to conventional drugs, and a QEPPI threshold of 0.5 is sufficient to identify approximately 75% of PPI-targeting compounds. Furthermore, PPI-target drugs have been removed from the FDA dataset based on the literature [21]; as there are few PPI-targeting compounds in the FDA dataset, the smaller QEPPI scores in the FDA dataset compared to those in the iPPI-DB dataset are consistent. However, the results in Figure 2 show that for each dataset, QED and QEPPI had almost opposite trends, and thus, 1−QED may be a similar index to QEPPI. Therefore, we calculated the ROC curve and area under the curve (AUC) to evaluate the quantitative performance of QEPPI and 1−QED in identifying PPI-targeting compounds. The true-positive rate and false-positive rate were calculated to plot the ROC curve. Figure 3 shows the ROC curves obtained from QEPPI, QED, and the value of 1−QED. For QED, the AUC was less than 0.5 (0.362), which was worse than that obtained using randomly selected compounds. This is consistent with the results shown in Figure 2a, which shows that the AUC of QEPPI (0.789) was higher than that of 1−QED (0.638), clearly indicating that QEPPI performs better than 1−QED in identifying whether a compound is likely to be a PPI-targeting compound. The FDA dataset removed 13 compounds that overlapped with PPI-targeting drugs in the “Truong approved dataset”. We also performed calculations using the dataset of 1609 compounds without removing the 13 PPI-targeting compounds, which gave an AUC of QEPPI, QED, and 1−QED values of 0.789, 0.365, and 0.635, respectively (see Supplementary Figure S1).

### 2.3. QEPPI Extends the Rule-of-Four

Morelli et al. proposed the “rule-of-four” (RO4) to evaluate PPI inhibitors [8]. This proposal was based on a statistical analysis of 39 PPI inhibitors in 2P2Idb [24] (currently, 2P2Idb is not accessible. Thus, we cannot use data from 2P2Idb). They calculated the general characteristics of the chemical space in which PPI inhibitors differed from FDA-approved drugs. As a result, the RO4 consists of the following four criteria for physicochemical properties:

- MW must be higher than 400;
- ALogP must be higher than 4;
- HBA must be higher than 4;
- The number of rings (RING) must be higher than 4.

Figure 3 shows that we could convert a discrete value index into a continuous value index, as the ROC curve of QEPPI and each point of RO4 were very close to each other. The result suggests that QEPPI is a general extension of the RO4 concept.

The threshold value of QEPPI can be adjusted. We calculated the threshold value of QEPPI (QEPPI scores with a threshold value of 0.5196) such that the F-score was maximized. We then used the iPPI-DB data as a positive sample and the FDA data as a negative sample to compare the discriminative performance of RO4, allowing one violation in the QEPPI. The confusion matrix and F-score results for RO4 and QEPPI are described in Table 2 and Table 3.

Table 3 shows that the F-score of QEPPI and RO4 are 0.501 and 0.451, respectively, indicating that QEPPI performs better than RO4. We performed the calculation again using the dataset of 1609 compounds without removing the 13 PPI-targeting compounds, obtaining the F-score of 0.499 and 0.446 for QEPPI and RO4, respectively (see Supplementary Figure S1).

**Figure 4:**
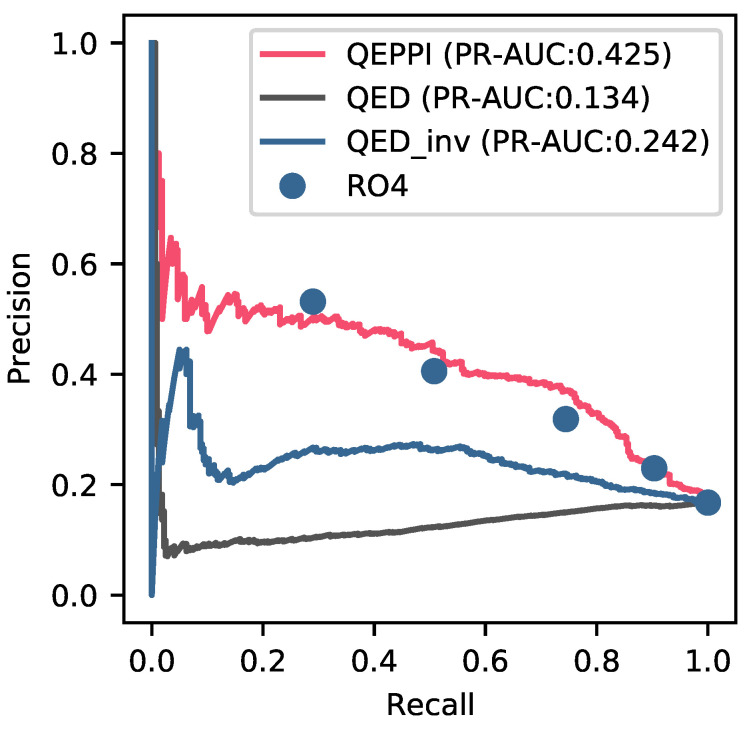
Comparison of QEPPI with RO4 in a Precision-Recall curve. The Precision-Recall curve shows Precision against the Recall value, which describes differences in performance for classifying compounds as PPI-target compounds. The red, black, and blue lines represent the Precision-Recall curves for QEPPI, QED, and 1−QED (QED_inv), respectively. The five blue dots represent the points that allowed 0 to 4 violations of RO4.

Finally, to compare the classification performance of two different metrics, namely, RO4 (rule-based) and QEPPI (threshold-based), we compared the value of Recall between the same value of Precision and value of Precision between the same value of Recall. The Precision-Recall curve is shown in Figure 4. Because RO4 is rule-based, we plotted the curves for all violations from one to four. As a result, each point of RO4, although not all RO4 points, was plotted on the lower side of the Precision-Recall curve of QEPPI. We also performed the calculation using the dataset of 1609 compounds without removing the 13 PPI-targeting compounds, resulting in AUC values for QEPPI, QED, and 1−QED values of 0.422, 0.134, and 0.238, respectively (see Supplementary Figure S2).

## 3. Discussion

### 3.1. Advantage of QEPPI

Theoretically, we represent the ideal values for each physicochemical property characteristic of that dataset. This is because the frequency of compounds with that property was highest in that dataset. Therefore, these properties are expected to reflect the nature of the target proteins. Furthermore, because QED is modeled using FDA-approved oral drugs, it is expected to reflect absorption, distribution, metabolism, excretion, and toxicity. In contrast, the dataset used for QEPPI involves many PPI-targeting compounds and does not involve any optimization. Hence, the peak values for all physicochemical properties were higher for QEPPI than those for QED.

The advantage of QEPPI is that it allows model building using only target data. It does not require appropriate negative samples. The performance of machine learning classifiers is poor in problem settings where positive and negative samples are imbalanced [25]. Therefore, QEPPI may be more effective than machine learning models under conditions in which appropriate negative samples are difficult to obtain from public databases.

RO4 is rule-based; therefore, it is nearly impossible to adjust certain threshold values. However, the threshold values of QEPPI developed in this study can be adjusted such that the desired sensitivity and specificity are achieved.

QEPPI indices are primarily intended to be used in the early-stage of PPI drug discovery, which is the seed compound discovery stage. Hence, better discrimination performance is desirable. Figure 4 shows that QEPPI has higher Precision at the same Recall and higher Recall at the same Precision than those for RO4 with one, two, and four violations of RO4. The Precision-Recall AUC values for QEPPI, QED (a measure of oral drug-like properties), and QED_inv (a measure of 1−QED) were 0.425, 0.134, and 0.242, respectively, indicating that among these measures, QEPPI most accurately identified PPI-targeting compounds. The rules of RO4 are based on only 39 PPI inhibitors and, as with RO5, the strict cutoff for each physicochemical property is controversial. For example, a molecular weight of 401 is a pass, whereas 399 is a violation. In fact, Table 1 shows that the peak value for MW is approximately 500 and peak values for ALogP and HBA are slightly higher than 4. This means that many compounds violate the RO4 criteria. For the 1007 PPI-targeting compounds used in the QEPPI model, the results of calculating whether each physicochemical property used in RO4 violates the four criteria are shown in Table 4. Table 4 shows that the violation percentages of WM, ALOP, and HBA were 24.1%, 37.5%, and 34.5%, respectively. For RING, which is a physicochemical property used only for RO4, more than 50% of the compounds violated this property.

The results above suggest that QEPPI is more useful and suitable compared to the conventional drug discovery indices QED and RO4. Hence our proposal is a useful index of PPI-targeting compounds in designing for early detection of PPI drugs.

Additionally, in further studies, QEPPI can be used as a reward in sequence-based molecular generation models using reinforcement learning such as REINVENT [18,19], and as a condition for sequence-based molecular generation models using conditional Wasserstein generative adversarial networks (WGANs) and Variational Autoencoders (VAEs), such as gcWGAN [26] and CVAE [27], which will enable molecular design with high PPI-targeting compound properties.

### 3.2. Application of QEPPI to PPI-Targeting Compounds That Are Approved or in Clinical Trials, and Other Small Compounds

In 2020, Shin et al. reported a review of PPI-targeting drug designs. We applied QEPPI to one dataset in this review [15]. The dataset is described as the non-PPI dataset in the review (Soga dataset) [28]. In 2021, Truong et al. explored which physicochemical parameters are necessary for a PPI modulator to become a clinical drug by analyzing the physicochemical properties of small-molecule PPI modulators that are either on the market, in clinical trials, or have been published. They found that PPI modulators currently on the market have a wide range of values for most physicochemical parameters, whereas PPI modulators in clinical trials conform much more closely to standard drug-like parameters, and therefore, a new PPI-specific screening library could be designed. This suggests that when designing new PPI-specific screening libraries, it is necessary to remain within parameters similar to those of standard drugs to obtain clinical candidates [21]. As suggested by the authors, PPI modulators undergoing clinical trials tend to have physicochemical properties more similar to those of standard drugs than those of PPI modulators currently on the market.

**Figure 5:**
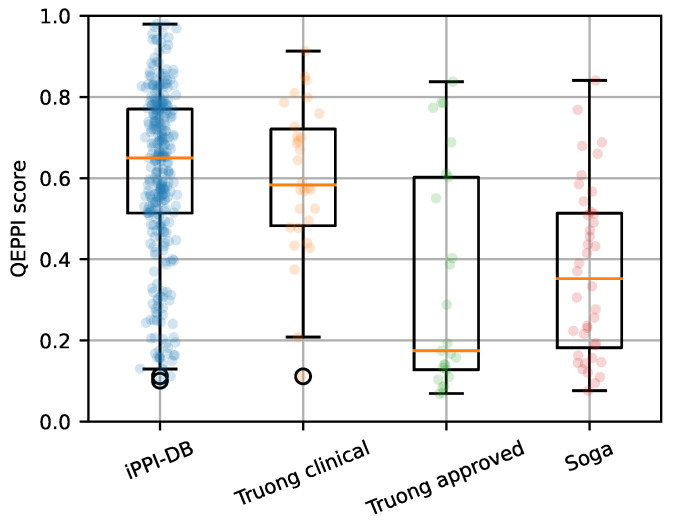
Distribution of QEPPI with respect to compounds in the clinical phase or approved PPI-targeting compounds dataset. The Truong clinical and Truong approved datasets represent clinical and FDA-approved PPI-targeting compound data, respectively. The iPPI-DB and Soga datasets represent positive and negative controls, respectively. The jitter overlaid on the boxplots shows the QEPPI scores for all samples in each data set.

We also applied QEPPI to the above datasets. The distribution of the QEPPI is shown in Figure 5. Our application of QEPPI to the 30 clinical candidates used by Truong et al. showed a median value of approximately 0.59, which is higher than that of commercially available PPI modulators, Figure 5. Although the physicochemical properties of the PPI-targeting compounds registered in iPPI-DB and FDA-approved drugs are different, as shown in Figure 1 and Table 1, the QEPPI modeled from iPPI-DB shows potential to be adapted to more recent PPI modulators.

**Figure 6:**
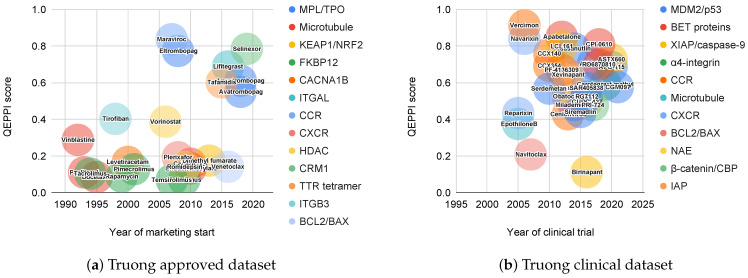
QEPPI scores for PPI-targeting marketed drugs within the past 30 years in Truong approved dataset and compounds in clinical trials within the past 30 years in Truong clinical dataset. QEPPI scores of PPI-targeting marketed drugs in Truong approved dataset (**a**), and QEPPI scores of PPI-targeting compounds in clinical trials in Truong clinical dataset (**b**). The color of the circle in each figure indicates the target for that drug or compound (in detail Supplementary Table S4).

In addition, we also looked at when the PPI-targeted compounds included in the Truong approved data were marketed and when the PPI-targeted compounds included in the Truong clinical data were used in clinical trials. Figure 6 shows the QEPPI of PPI-targeting marketed drugs and compounds in clinical trials within the last 30 years (in detail Supplementary Table S4). Figure 6a shows the PPI-targeting drugs on the market, year the drug was first marketed (as identified in DrugBank), QEPPI value, and target PPI for each drug. PPI-targeting drugs launched in the 1990s showed lower QEPPI scores, whereas drugs marketed more recently tended to have higher QEPPI scores. Figure 6b shows the PPI-targeting compounds in clinical trials, year of the first clinical trial (identified in ClinicalTrials.gov, accessed on 15 September 2021), EU Clinical Trials Register (or NIPH Clinical Trial Search in Japan), QEPPI value, and target PPI for each compound. Regardless of the year, the QEPPI scores showed a high transition. This is consistent with the fact that the QEPPI scores of the marketed drugs in Figure 6a exhibited a recent trend toward higher values.

In this study, some PPI-targeting compounds with low QEPPI scores showed small molecular weights compared to those at the peak. A previous study showed that the size and complexity of the binding interface of PPIs varies depending on the target. If the interface is relatively less complex and small, some PPI-targeting compounds with relatively small molecular weights can sufficiently block the binding interface. When the binding interface is more complex, the binding interface tends to be wide, and only a PPI-targeting compound with a large molecular weight can sufficiently block the binding interface [29].

**Figure 7:**
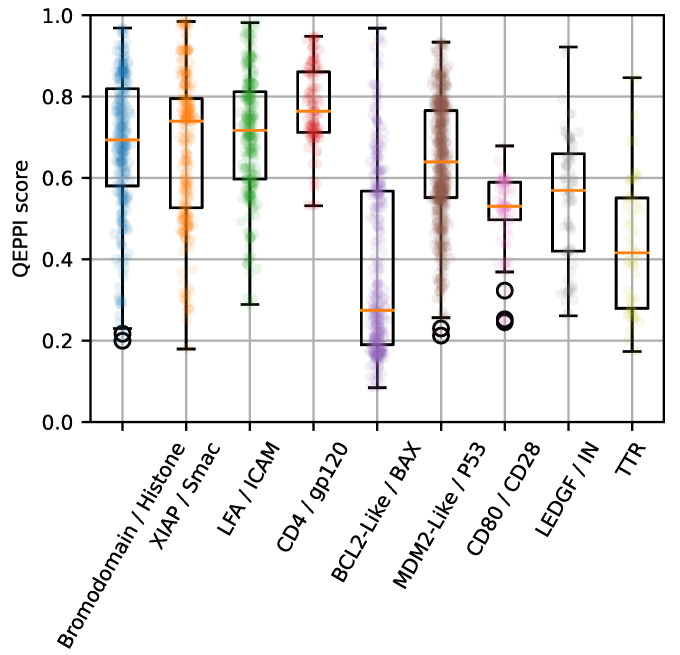
Distribution of QEPPI scores for 9 PPI families with more than 50 compounds targeting each PPI family in iPPI-DB. The jitter overlaid on the boxplots shows the QEPPI scores for all samples in each dataset. Statistics related to this figure are shown in Supplementary Table S5.

Figure 7 shows that there is a difference in the distribution of QEPPI for each PPI family in the iPPI-DB dataset. This result shows that the QEPPI scores of compounds targeting Bromodomain/Histone [29], XIAP/Smac [29], LFA/ICAM [29], and CD4/gp120 [30], which have primary epitopes (such as linear peptide), tend to be higher than those of compounds targeting Bcl2/Bax [29], p53/MDM2 [29], and CD80/CD28 [31], which have secondary epitopes (such as the helix structure). As the LEDGF/IN interface area (400 Å) and transthyretin (TTR) dimer-dimer interface area are much smaller than the interface area of other PPIs [15,29,32], the QEPPI scores of these PPI-targeting compounds tended to be low. Thus, the difference in the complexity of the PPI interface may affect the physicochemical properties of PPI-targeting compounds such as molecular weight; furthermore, the complexity of the PPI interface is related to the QEPPI score.

Therefore, evaluating average PPI-targeting compounds using the iPPI-DB as a dataset of various types of PPI-targeting compounds would be advantageous. Our further studies will focus on designing indices that are more specific to PPI-targeting compounds, such as the size of the binding interface or PPI family. This is similar to the proposal of QEX. The approach will become feasible as more data are deposited in the database.

Various types of PPI-targeting compounds have been reviewed, and QEPPI scores were calculated for several types of PPI target compounds mentioned in the review published by Mabonga et al. [33]. For example, nine compounds that target chemokine receptors were included in the Truoung approved and Truoung clinical datasets used in this study, with an average QEPPI score of 0.641 (see Supplementary Table S6). In addition, compounds targeting MDM2/p53, a cancer-related PPI, were also included in the Truoung clinical dataset with an average QEPPI score of 0.593 (see Supplementary Table S6). The average QEPPI scores of 79-6 (PubChem CID5721353) targeting BCL6/SMRT and FMP-API-1 targeting AKAP18δ/PKA were 0.468 and 0.410, respectively (see Supplementary Table S7). In addition, LFA/ICAM, a PPI related to T-cell activation, was included in the data downloaded from iPPI-DB, and the average QEPPI score was 0.706 (see Supplementary Table S5). These results suggest that QEPPI is effective for PPI modulators that have been developed to date. However, the QEPPI scores of approved drugs targeting FKBP12 (e.g., pimecrolimus, tacrolimus, everolimus, rapamycin, temsirolimus) and approved drugs targeting microtubules with a molecular weight greater than 800 (cabazitaxel, docetaxel cabazitaxel, eribulin mesylate, paclitaxel, vinblastine) have lower QEPPI scores (see Supplementary Table S6). This is because the iPPI-DB does not include macrocyclic compounds that target FKBP12, and only approximately 4% of the total iPPI-DB of compounds have molecular weights exceeding 800; the QEPPI score of such compounds that deviate from the average is considered low because of the nature of the method. Further studies are needed to expand the compound space that is not covered by iPPI-DB.

To date, COVID-19 has claimed the lives of more than 4.7 million people and infected another 230 million, making it a global pandemic. In response to this critical situation, the development of drugs targeting the etiological agent, SARS-CoV-2, is ongoing. Some researchers are focusing on compounds that target PPIs. One of the most promising PPI targets is the interaction between the SARS-CoV-2 S protein and human angiotensin-converting enzyme 2 receptor [34,35,36]. Therefore, we also calculated QEPPI for small-molecules targeting PPIs against SARS-CoV/SARS-CoV-2 (see Supplementary Table S8). The median QEPPI score for these compounds was 0.511. Although this is only an example, QEPPI may be effective for host-pathogen PPIs and other PPIs.

### 3.3. Limitations and Challenges

In this study, the physicochemical properties of the structural alerts were not used in the model building process. As mentioned in the methods, because of the nature of the database, the ratio of compounds in iPPI-DB that do not contain structural alerts is higher than the ratio of compounds in oral FDA-approved drugs which were used in QED that do not contain structural alerts. Therefore, addition of physicochemical properties of ALERTS may bias the model to give undue preference to compounds with low ALERTS values. In the early stages of the actual screening process, it is necessary to filter out structural alerts such as highly reactive functional groups. For example, it is possible to filter out compounds with an “unwanted group” structure described in the supplementary material of the paper published by Brenk et al. [37], which was also used in QED. It is also necessary to filter out other artifacts that do not depend on specific drug-like interactions between molecules and proteins, known as PAINS (pan assay interfering compounds) [38]. During actual operation, these filters should be used in conjunction with the QEPPI score in screening.

In the present QEPPI model, we focused on the physicochemical properties and did not consider the stereo-coordination of the compounds targeting PPI. However, it has been suggested that 3D conformation properties are important for PPI-targeted compounds [29]. It is likely that many PPI-targeted compounds are large and have a high degree of freedom in their bond angles, which may also be characteristic of their stereo-coordination. Therefore, the investigation of methodologies that consider stereo-coordination is one of the important issues to be addressed in the future.

## 4. Materials and Methods

### 4.1. Calculation of QEPPI

QEPPI was calculated using essentially the same procedure as used for the original QED, except that it was modeled using compounds curated in the iPPI-DB. We did not use ‘ALERTS’ among the physicochemical properties, because approximately 58.7% of compounds in the iPPI-DB do not contain a substructure on the list of structural alerts and 22.6% of compounds had only one substructure on the list of structural alerts. Because of the nature of the database, the percentage of compounds without structural alerts is large and may create biases if they are added to the physicochemical properties of the modeling, and thus, these compounds were excluded from the physicochemical properties during modeling. The algorithms used are described below. In the first modeling step, RDKit (2020.09.1) was used to calculate seven molecular physicochemical properties: the molecular weight (MW), LogP value estimated by the Wildman-Crippen method [39] (ALogP), number of hydrogen bond donors (HBD), number of hydrogen bond acceptors (HBA), topological molecular polar surface area (TPSA), number of rotatable bonds (ROTB), and number of aromatic rings (AROM). Table 5 lists the RDKit functions used to calculate these properties.

A histogram of each property was created and fitted to the asymmetric double sigmoid function Q(x) shown in Equation (1) by implementing the Levenberg-Marquardt algorithm in SciPy (version 1.6.1).

(1) $$\begin{array}{c} Q\left(x\right)=a+\frac{b}{1+\exp\left(-\frac{x-c+\frac{d}{2}}{e}\right)}\left[1-\frac{b}{1+\exp\left(-\frac{x-c-\frac{d}{2}}{f}\right)}\right] \end{array}$$

All fitting functions ($Q_{\mathrm{MW}}\left(x\right)$, $Q_{\mathrm{ALogP}}\left(x\right)$, $Q_{\mathrm{HBD}}\left(x\right)$, $Q_{\mathrm{HBA}}\left(x\right)$, $Q_{\mathrm{TPSA}}\left(x\right)$, $Q_{\mathrm{ROTB}}\left(x\right)$, and $Q_{\mathrm{AROM}}\left(x\right)$) were divided by the maximum value and normalized to a maximum value of 1. The normalized function ${\tilde{Q}}_{i}\left(x\right)\left(i\in \left\{\mathrm{MW},\mathrm{ALogP},\mathrm{HBD},\mathrm{HBA},\mathrm{TPSA},\mathrm{ROTB},\mathrm{AROM}\right\}\right)$ was used as the desirability function. Finally, the QEPPI score of compound *k* was assigned as the weighted geometric mean of all desirability functions [40], as shown in Equation (2).

(2) $$\begin{array}{c} {\mathrm{QEPPI}}_{k}=\exp\left(\frac{\sum_{i}w_{i}\ln\left({\tilde{Q}}_{i}\right)}{\sum_{i}w_{i}}\right) \end{array}$$

The seven weights were tested from 0 to 1 in increments of 0.25, and the average of the 1000 combinations of weights resulting in the highest Shannon entropy was adopted. The Shannon entropy of the model was calculated as shown in Equation (3), where *n* represents the number of compounds used for modeling.

(3) $$\begin{array}{c} \mathrm{entropy}=-\sum_{k=1}^{n}{\mathrm{QEPPI}}_{k}\log_{2}{\mathrm{QEPPI}}_{k} \end{array}$$

### 4.2. Calculation of QED and RO4

To evaluate the filtering performance of QEPPI, QED and RO4 were calculated and used for comparison. The QED score was calculated using the Chem.QED.qed method in RDKit. RO4 is calculated from four properties: the MW, ALogP, HBA, and number of ring structures (RING). MW, ALogP, and HBA were calculated using the same methods as those used for QEPPI. RING was calculated using the Chem.rdMolDescriptors.CalcNumRings method in RDKit (Table 5).

### 4.3. Dataset

To create a non-redundant dataset for the QEPPI model, we downloaded 2361 SMILES and other data of compounds registered in iPPI-DB, and 1007 compounds were selected from all clusters individually with the best activities determined by clustering with Bemis-Murcko atomic frameworks [41].

- As a dataset for evaluating QEPPI, 321 compounds were selected from all clusters individually with the best activities from all clusters of compounds that were not used for model building (iPPI-DB dataset).
- As a dataset for small-molecule compounds, we obtained SMILES and other data of compounds known as “DrugBank FDA only” compounds from the catalog of ZINC [42] and removed duplicates by InChI, resulting in 1609 compounds. In addition, 13 compounds that overlapped with the “Truong approved dataset” PPI-targeting drugs (as explained below) were removed, resulting in 1596 compounds (FDA dataset).
- As a dataset for PPI-targeting compounds that are either in the clinical stage or have been approved, 30 PPI-targeting compounds in clinical trial stages and 25 FDA-approved PPI-targeting compounds were obtained from [21] (Truong clinical and Truong approved datasets, respectively).
- As a dataset of non-PPI ligands, which involved known non-redundant protein-ligand complexes evaluated using X-ray crystallography, we obtained 40 PDB IDs of single-molecule ligands obtained from [28] (Soga dataset).

For datasets for which only PDB IDs were available, the IDs were converted to SMILES using PDB’s GraphQL-based API [43].

### 4.4. Performance Measures

Using the “iPPI-DB dataset” as the positive dataset and “FDA dataset” as the negative dataset, samples scoring above a certain threshold by QEPPI or QED were predicted as positive, and samples scoring below the threshold were predicted as negative. The performance measures used are shown in Equations (4) and (5):

(4) $$\begin{array}{cc} \mathrm{TPR}=\mathrm{Recall} & =\frac{\mathrm{TP}}{\mathrm{TP}+\mathrm{FN}} \end{array}$$

(5) $$\begin{array}{cc} \mathrm{FPR} & =\frac{\mathrm{FP}}{\mathrm{FP}+\mathrm{TN}} \end{array}$$

where TP, FP, FN, TN, TPR, and FPR are the numbers of true-positives, false-positives, false-negatives, true-negatives, and true-positive and false-positive ratios, respectively.

Furthermore, the F-score shown in Equation (6) was used to evaluate the discrimination performance, and the precision shown in Equation (7) was used for the Precision-Recall curve (Recall is already shown in Equation (4)).

(6) $$\begin{array}{cc} F-\mathrm{score} & =\frac{2\cdot \mathrm{TP}}{2\cdot \mathrm{TP}+\mathrm{FP}+\mathrm{FN}} \end{array}$$

(7) $$\begin{array}{cc} \mathrm{Precision} & =\frac{\mathrm{TP}}{\mathrm{TP}+\mathrm{FP}} \end{array}$$

To evaluate the receiver operating characteristic (ROC) and Precision-Recall curves, the QEPPI threshold was calculated based on all QEPPI scores in the data set, TP, FP, FN, and TN. In Table 6, the threshold was calculated, in which the F-score was maximized.

## 5. Conclusions

QEPPI is based on the concept of QED, which models the physicochemical properties of a target compound and can quantify the PPI-targeting drug-likeness of interest compounds using PPI inhibitors and stabilizers as the target compound. The metric is useful in the early detection of PPI drugs.

RO4 was originally proposed as a rule-based approach with respect to a statistical analysis of the physicochemical characteristics of actual PPI inhibitors. QED is also based on the distribution data of the physicochemical properties of oral drugs and has gained attention in early-stage drug discovery. However, this method is not suitable for early-stage screening of PPI-targeting compounds because the physicochemical properties of PPI-targeting compounds differ significantly from those of oral drugs. In addition, compared to the rule-based approach of RO4, QEPPI is based on the basic distribution data of physicochemical properties of more PPI-targeting compounds. Unlike rule-based indices, when many parameters of physicochemical properties are ideal, certain unfavorable parameters of properties may still be acceptable, making it an extremely useful index, specifically for early-stage screening of compounds targeting PPIs.

QEPPI may lead to the development of PPI-based drugs along with consequent improvements in the accuracy of QEPPI as more PPI-targeting compounds are registered in the database.

## Figures and Tables

**Table 1:** Distribution peaks and optimized desirability function weightings of each molecular physicochemical property.

		MW	ALogP	HBD	HBA	TPSA	ROTB	AROM
peak	QED *	305.8	2.70	1.20	2.38	57.5	3.04	1.8
	QEPPI	492.7	4.78	1.61	4.79	76.9	6.37	2.8
$w_{i}$	QED *	0.66	0.46	0.61	0.05	0.06	0.65	0.48
	QEPPI	0.47	0.10	0.82	0.81	0.37	0.53	0.89

* QED was modeled as a function that includes ALERTS; the peak value of ALERTS in QED was −24.6, and its weight $w_{\mathrm{ALERTS}}$ was 0.95.

**Table 2:** Confusion matrix based on RO4 with one violation.

	Passed	Failed
positive	163	158
negative	239	1357

**Table 3:** Precision, Recall, and F-score values for one violation of RO4 and QEPPI scores with a threshold value of 0.5196.

	Precision	Recall	F-Score
RO4	0.405	0.508	0.451
QEPPI	0.379	0.735	0.501

**Table 4:** RO4 violations in the dataset used for QEPPI modeling.

	MW	ALOGP	HBA	RING
violation	243	378	347	532
no violation	764	629	660	475
violation rate	0.241	0.375	0.345	0.528

**Table 5:** RDKit functions used to calculate the molecular properties used in quantitative estimate of protein-protein interaction targeting drug-likeness (QEPPI) and rule-of-four (RO4).

Property	RDKit Function
MW	Chem.rdMolDescriptors.CalcExactMolWt
ALogP	Chem.Crippen.MolLogP
HBD	Chem.rdMolDescriptors.CalcNumHBD
HBA	Chem.rdMolDescriptors.CalcNumHBA
TPSA	Chem.rdMolDescriptors.CalcTPSA
ROTB	Chem.rdMolDescriptors.CalcNumRotatableBonds
AROM	Chem.rdMolDescriptors.CalcNumAromaticRings
RING	Chem.rdMolDescriptors.CalcNumRings

**Table 6:** Confusion matrix based on QEPPI scores with a threshold value of 0.5196.

	Passed	Failed
positive	236	85
negative	386	1210

## Data Availability

The compound data sets used in this study, implementation code, and easy-to-use environment on Google Colaboratory are available under the MIT license at https://github.com/ohuelab/QEPPI, accessed on 30 September 2021.

## Supplementary Materials

The following are available online at https://www.mdpi.com/article/10.3390/ijms222010925/s1.

## Abbreviations

The following abbreviations are used in this manuscript:

QED	Quantitative estimate of drug-likeness
PPI	Protein-protein interaction
QEPPI	Quantitative estimate index for compounds targeting protein-protein interactions
RO4	Rule of four
RO5	Rule of five
PPI	Protein-protein interaction
FDA	The U.S. Food and Drug Administrations
MW	Molecular weight
ALogP	LogP value estimated by the Wildman-Crippen method
HBD	The number of hydrogen bond donors
HBA	The number of hydrogen bond acceptors
TPSA	Topological molecular polar surface area
ROBT	The number of rotatable bonds
AROM	The number of aromatic rings
RING	The number of ring structures
ALERTS	The number of structural alerts
ROC	Receiver operating characteristic
AUC	Area under the curve
WGAN	Wasserstein generative adversarial network
VAE	Variational Autoencoder
TTR	Transthyretin
PAINS	Pan assay interfering compounds
