# Supplementary material for: Quantitative Estimate Index for Early-Stage Screening of Compounds Targeting Protein-Protein Interactions
Source: Int J Mol Sci. 2021 Oct 10;22(20):10925. doi: 10.3390/ijms222010925 (PMC8539639; doi:10.3390/ijms222010925)
Supplement: Supplementary file 1 [file ijms-22-10925-s001.zip › ijms-1398555-Sup-for proof-authoredited.pdf]

Supplementary Materials

Quantitative Estimate Index for Early-Stage Screening of Compounds Targeting Protein-Protein Interactions

Takatsugu Kosugi and Masahito Ohue*

Department of Computer Science, School of Computing, Tokyo Institute of Technology
* Correspondence: ohue@c.titech.ac.jp; Tel.: +81-45-924-5522

1. SUPPLEMENTARY FIGURES

These figures are receiver operating characteristic (ROC) and Precision-Recall curves for quantitative estimate of protein-protein interaction targeting drug-likeness (QEPPI), $1 - \text{QED}$, and quantitative estimate of drug-likeness (QED) calculated using the dataset of 1609 compounds before the removal of 13 protein-protein interaction (PPI) target compounds when creating the FDA dataset. (See Methods)

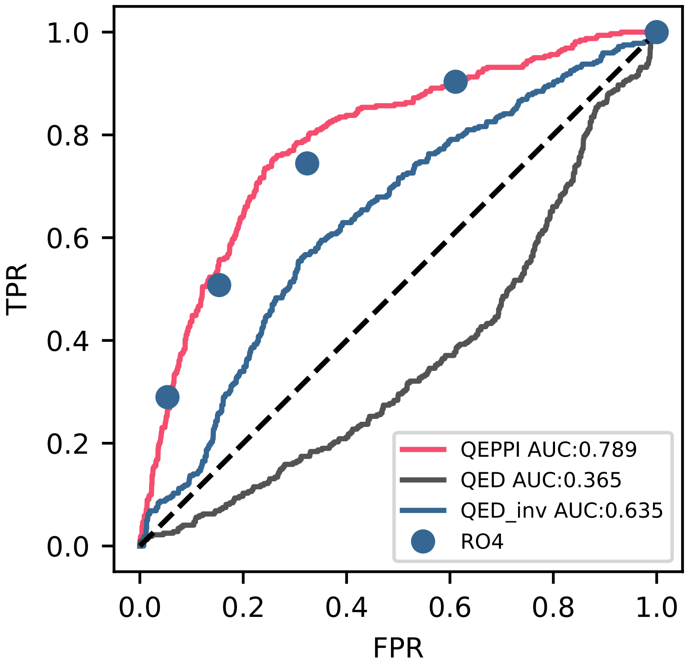

Figure. S1. Comparison of quantitative estimate of protein-protein interaction targeting drug-likeness (QEPPI) with other measures of drug-like properties in receiver operating characteristic (ROC) curves using a dataset of 1609 compounds without the removal of 13 approved protein-protein interaction (PPI)-targeting drugs from the FDA dataset. All ROC curves show that the true positive rate against the false positive rate describes the differences in performance for classifying compounds as PPI-targeting compounds. The red, black, and blue lines represent the ROC curves for QEPPI, quantitative estimate of drug-likeness (QED), and $1 - \text{QED}$ (QED_inv), respectively. The five blue dots are plotted as points that allowed 0 to 4 violations of Rule-of-Four (RO4). The dashed black line represents a random prediction of the dataset.

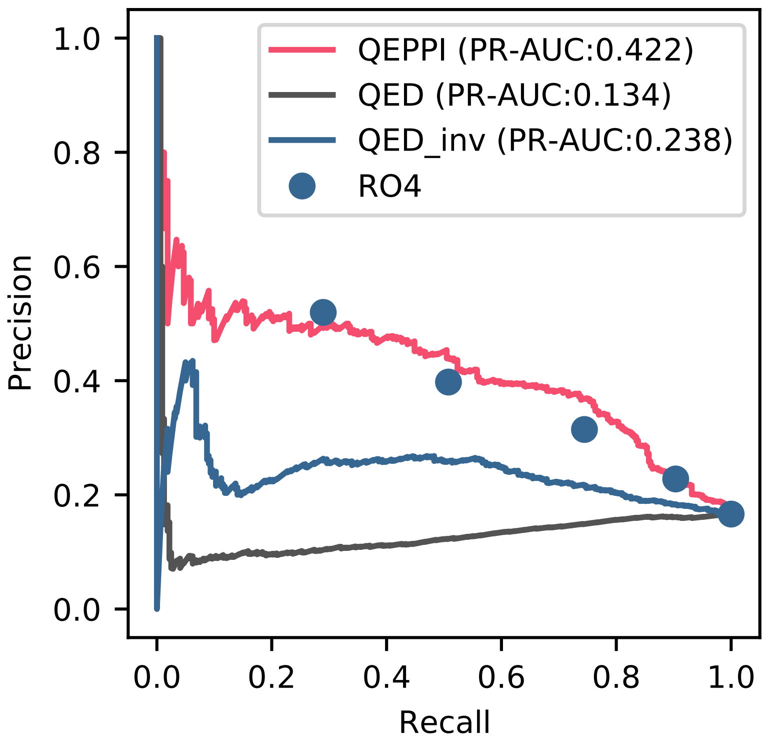

Figure. S2. Comparison of QEPPI with RO4 in Precision-Recall curve using a dataset of 1609 compounds without the removal of 13 approved PPI-targeting drugs from the FDA dataset. The Precision-Recall curve shows the precision against the recall value, which describes the differences in performance for classifying compounds as PPI-targeting compounds. The red, black, and blue lines represent the Precision-Recall curves for QEPPI, QED, and $1 - \text{QED}$ (QED_inv), respectively. The five blue dots represent the points that allowed 0 to 4 violations of RO4.

2. SUPPLEMENTARY TABLES

These tables are Confusion matrices, Precision, Recall, and F-score based on RO4 and QEPPI calculated using a dataset of 1609 compounds before removing 13 PPI target compounds when creating the FDA dataset. (See Methods)

Table S1. Confusion matrix based on RO4 with one violation.

	passed	failed
positive	163	158
negative	247	1,362

Table S2. Confusion matrix based on QEPPI scores with a threshold value of 0.5196.

	passed	failed
positive	236	85
negative	389	1,220

Table S3. Precision, Recall, and F-score values for one violation of RO4 and QEPPI scores with a threshold value of 0.5196.

	Precision	Recall	F-score
RO4	0.398	0.508	0.446
QEPPI	0.378	0.735	0.499
